# tRF-Gly-GCC in Atretic Follicles Promotes Ferroptosis in Granulosa Cells by Down-Regulating MAPK1

**DOI:** 10.3390/ijms25169061

**Published:** 2024-08-21

**Authors:** Yuheng Pan, Mailin Gan, Shuang Wu, Yuxu He, Jinkang Feng, Yunhong Jing, Jiaxin Li, Qian Chen, Jiang Tong, Lingfan Kang, Lei Chen, Ye Zhao, Lili Niu, Shunhua Zhang, Yan Wang, Li Zhu, Linyuan Shen

**Affiliations:** 1 State Key Laboratory of Swine and Poultry Breeding Industry, College of Animal Science and Technology, Sichuan Agricultural University, Chengdu 611130, China; panyuheng@stu.sicau.edu.cn (Y.P.); ganmailin@sicau.edu.cn (M.G.); wushuang@stu.sicau.edu.cn (S.W.); heyuxu@stu.sicau.edu.cn (Y.H.); fengjinkang@stu.sicau.edu.cn (J.F.); jingyunhong@stu.sicau.edu.cn (Y.J.); lijiaxin1@stu.sicau.edu.cn (J.L.); chenqian1@stu.sicau.edu.cn (Q.C.); 202100216@stu.sicau.edu.cn (J.T.); kanglingfan@stu.sicau.edu.cn (L.K.); chenlei815918@sicau.edu.cn (L.C.); zhye@sicau.edu.cn (Y.Z.); niulili@sicau.edu.cn (L.N.); 14081@sicau.edu.cn (S.Z.); wangyan2023@sicau.edu.cn (Y.W.); 2 Key Laboratory of Livestock and Poultry Multi-Omics, Ministry of Agriculture and Rural Affairs, College of Animal Science and Technology, Sichuan Agricultural University, Chengdu 611130, China; 3 Farm Animal Genetic Resources Exploration and Innovation Key Laboratory of Sichuan Province, Sichuan Agricultural University, Chengdu 611130, China

**Keywords:** atretic follicle, granular cell, ferroptosis, tRF-1:30-Gly-GCC-2, *MAPK1*

## Abstract

Follicle development refers to the process in which the follicles in the ovary gradually develop from the primary stage to a mature state, and most primary follicles fail to develop normally, without forming a dense granular cell layer and cell wall, which is identified as atretic follicles. Granulosa cells assist follicle development by producing hormones and providing support, and interference in the interaction between granulosa cells and oocytes may lead to the formation of atretic follicles. Ferroptosis, as a non-apoptotic form of death, is caused by cells accumulating lethal levels of iron-dependent phospholipid peroxides. Healthy follicles ranging from 4 to 5 mm were randomly divided into two groups: a control group (DMSO) and treatment group (10 uM of ferroptosis inducer erastin). Each group was sequenced after three repeated cultures for 24 h. We found that ferroptosis was associated with atretic follicles and that the in vitro treatment of healthy follicles with the ferroptosis inducer erastin produced a phenotype similar to that of atretic follicles. Overall, our study elucidates that tRF-1:30-Gly-GCC-2 is involved in the apoptosis and ferroptosis of GCs. Mechanistically, tRF-1:30-Gly-GCC-2 inhibits granulosa cell proliferation and promotes ferroptosis by inhibiting Mitogen-activated protein kinase 1 (*MAPK1*). tRF-1:30-Gly-GCC-2 may be a novel molecular target for improving the development of atretic follicles in ovarian dysfunction. In conclusion, our study provides a new perspective on the pathogenesis of granulosa cell dysfunction and follicular atresia.

## 1. Introduction

In recent years, improving the reproductive performance of *sows* is one of the research focuses, among which improving follicle quality and ovulation rate is the main research direction [1]. Even if atresia is a normal physiological process for selecting ovulation, it is sometimes inappropriate, too intensive, and many cases require improved knowledge in breeding and medicine. Mammalian ovaries are an important part of the reproductive system of female animals. They perform important functions such as endocrine regulation, oocyte formation and release, so they have been widely studied. The development of the ovary goes through multiple stages. During embryonic development, the germ cells in the embryo develop into the ovarian primordia. During embryonic development, the ovarian primordia differentiate into follicles, which do not fully develop in the fetal period but lay the foundation for future ovarian development [2]. When a person enters puberty, significant changes occur in the reproductive system. Increased levels of hormones, especially estrogen and luteinizing hormone (LH) and follicle-stimulating hormone (FSH), stimulate follicles in the ovaries to start developing [3]. At this stage, the follicles in the ovaries gradually mature in preparation for ovulation.

Follicular development refers to the gradual development of follicles in the ovaries from the primary stage to a mature state [4]. Gougeon A. conducted a study on a large number of whole follicle populations of normal ovaries in three stages: the initiation of follicle growth, growth promoting period, and gonadotropin-dependent period [5]. After summarizing the stages of follicle development, it was found that follicle development includes four stages: primordial follicles, primary and secondary follicles, and mature follicles. The primordial follicle is the initial stage of follicle development, characterized by the formation of multiple layers of cells around the oocyte, forming a pseudo-granular cell structure. Further maturation causes some primary follicles to develop into secondary follicles. At this stage, there is a continued proliferation of granular cells forming intricate structures while fluid accumulation commences within the expanding follicle. A portion of the secondary follicle develops into a mature follicle, which is regulated by hormones (especially luteinizing hormone) to rupture and release the egg into the fallopian tubes, a process known as ovulation [6]. This process usually involves apoptosis and other degenerative changes that eventually lead to complete degeneration, absorption, or disappearance. And this degenerative process is necessary to maintain the normal function of the reproductive system.

Ferroptosis is a special form of cell death. It is closely related to the abnormal accumulation of free iron ions in cells, oxidative stress, and lipid peroxidation [7]. Compared to other forms of cell death, such as apoptosis and necrosis, ferroptosis has unique biological characteristics. Its essence is the depletion of glutathione (GSH); when glutathione peroxidase (*GPX4*) activity is decreased, lipid oxides cannot be metabolized through the GPX4-catalyzed glutathione reductase reaction, and then the bivalent ferrous ions (Fe^2+^) oxidize the lipids to produce reactive oxygen species (ROS), thus promoting the occurrence of ferroptosis [8]. One characteristic of ferroptosis is the excessive accumulation of free iron ions in cells. This may be due to an abnormal iron metabolism, iron regulatory protein dysfunction, and other reasons. The accumulation of free iron ions triggers oxidative stress, resulting in the production of highly reactive ROS. This will damage the cell’s biofilm, lipid, and other structures. Ferroptosis is mainly a process of oxidative damage and antioxidation [7]. As a key step, lipid peroxidation refers to the destruction of unsaturated fatty acids in lipid molecules by oxygen free radicals to form lipid peroxidation. Due to lipid peroxidation, the cell membrane is destroyed, resulting in the imbalance of the internal and external environment of the cell. This can cause metabolic confusion within the cell, loss of ions, and loss of other cellular functions. Recent evidence suggests that ferroptosis may be associated with the development of female reproductive diseases, including polycystic ovary syndrome (PCOS), premature ovarian failure (POI), endometriosis (EMs), and ovarian cancer (OC) [9]. There are relatively few reports of ferroptosis in female mammalian reproductive diseases. It has been reported that in patients with endometriosis, excessive oxidative stress in granular cells leads to cellular aging and mitochondrial abnormalities [10]. The pathways and genes associated with ferroptosis may be involved in the regulation of granulosa cell proliferation and secretion, oocyte development, ovarian reserve function, early embryonic development, and placental oxidative stress [9].

Advances in RNA-seq technology have helped to identify small non-coding RNAs with lengths of approximately 13–48 nt. Further data showed that the abundance of this type of RNA was second only to miRNA, and it was precisely processed from the 5′ end or 3′ end of mature tRNA or pre-tRNA, resulting in several types of tRNA-derived small molecules [11]. With the widespread application of high-throughput RNA sequencing (RNA-seq), tRNA-derived small RNA (tsRNA) (also known as tDR or tRNA-derived fragments, tRF) is increasingly recognized as an emerging class of functional small non-coding RNAs [12]. tRF-1s, tRF-3s, and tRF-5s are included.

But so far, there are few reports on the regulation of physiological processes by tRNA. There are few reports that suggest that tRNA plays a role by mediating ferroptosis. This study aimed to investigate the role of tRF-1:30-GLy-GCC-2 in the pathogenesis of ferroptosis. We selected 4~5 mm healthy porcine follicles and randomly divided them into two groups: a control group (DMSO) and treatment group (10 uM ferroptosis inducer erastin). The culture was repeated three times in each group, and the expression of tRF-1:30-GLy-GCC-2 was significantly increased by sequencing 24 h later. Subsequently, tRF-1:30-GLy-GCC-2 was overexpressed on granulosa cells for validation in vitro. Finally, we speculated that tRF-1:30-GLy-GCC-2 might regulate the expression of MAPK1, thereby affecting the iron sagging of granulosa cells and possibly causing follicular atresia.

## 2. Results

### 2.1. The Occurrence of Ferroptosis in Sows during Follicular Atresia

**Figure 1:**
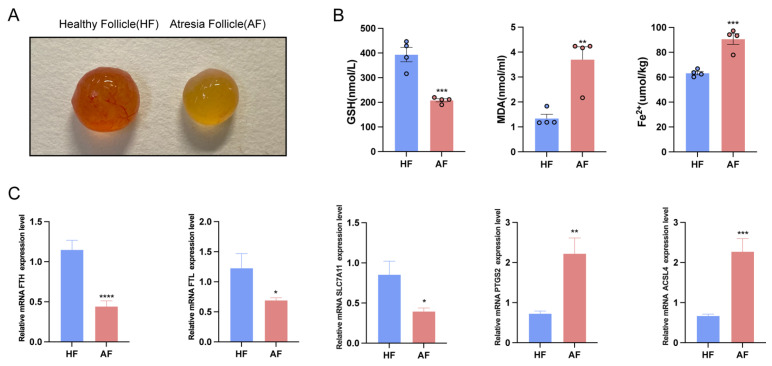
Ferroptosis occurs in atretic follicles. (**A**) Comparison of phenotypes between healthy follicles and atretic follicles. (**B**) The content of GSH, MDA, and Fe^2+^ in healthy and atretic follicles. (**C**) To further confirm the effect of ferroptosis on follicular development, we detected the down-regulated standard genes *FTH1*, *FTL*, and *SLC7A11* and the up-regulated marker genes *PTGS2* and *ACSL4* in follicles by qRT-PCR. Data represented the mean ± SEM of at least three biological replicates. * *p* < 0.05, ** *p* < 0.01, *** *p* < 0.001 and **** *p* < 0.0001, compared to control.

It has been reported that there are differences in the expression levels of glutathione and iron metabolism marker genes between healthy and atretic follicles, and some studies have suggested that the atresia of porcine follicles may be associated with the ferroptosis of cells. Compared with healthy follicles, the surface of atretic follicles is collapsed, the color is grayish, the transparency is low, and some follicle contents are cloudy (Figure 1A). In addition, we measured the levels of reduced GSH, malondialdehyde (MDA), and Fe^2+^ in the fluid of four healthy and four atretic follicles. The results showed that the GSH content in atretic follicles was significantly reduced compared to healthy follicles. The content of MDA in the follicular fluid of atretic follicles was significantly higher than that of healthy follicles, and the content of Fe^2+^ was significantly increased (Figure 1B). To explore the association between follicular atresia and ferroptosis, we examined the expression levels of iron metabolism marker genes in the follicular fluid of healthy and atresia follicles and found that the expression levels of Ferritin Heavy Chain 1 (*FTH*), Ferritin light chain (*FTL*), and Solute carrier family 7, membrane 11 (*SLC7A11*) in atresia follicles were significantly lower than those in healthy follicles. The expression levels of Prostaglandin-endoperoxide synthase 2 (*PTGS2*) and long-chain acyl-CoA synthetase (*ACSL4*) were significantly higher than those of healthy follicles (Figure 1C). These results indicate that the process of porcine follicle atresia is accompanied by ferroptosis, and ferroptosis may play an important regulatory role in the process of follicle atresia.

### 2.2. Ferroptosis Inducers Inhibit Granulosa Cell Proliferation and Promote Ferroptosis of Granulosa Cell

**Figure 2:**
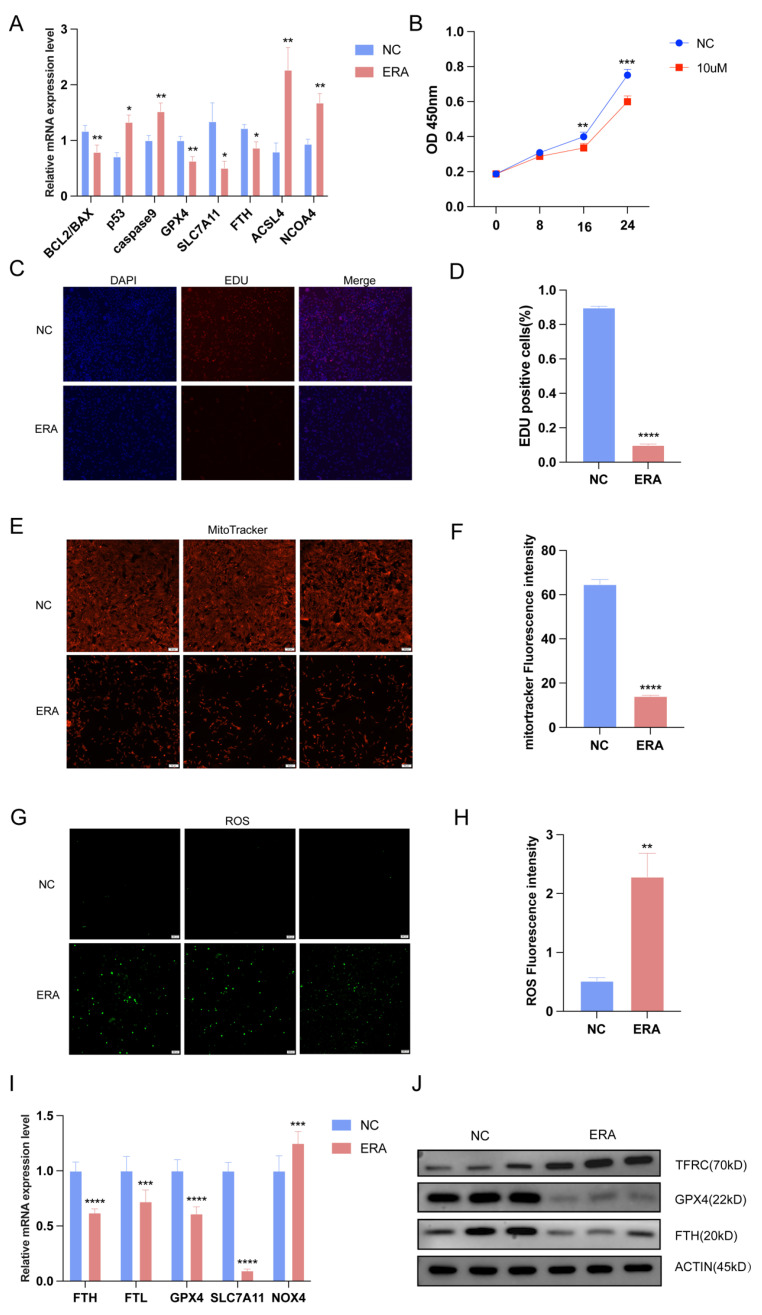
Ferroptosis inducers induced follicular atresia and granular cell ferroptosis in erastin. (**A**) The expression of ferroptosis marker genes were detected by qRT-PCR in erastin-treated follicles. The result of CCK-8 (**B**) is consistent with the result of EDU experiment (**C**,**D**). Mitotracker (50 μm) staining is used to detect mitochondrial activity in control group and erastin group (**E**,**F**); ROS (200 μm) staining is used to detect reactive oxygen species in control group and erastin group (**G**,**H**). Erastin induced ferroptosis in granular cells, and qRT-PCR (**I**) results are consistent with WB (**J**). Data represented the mean ± SEM of at least three biological replicates. * *p* < 0.05, ** *p* < 0.01, *** *p* < 0.001 and **** *p* < 0.0001, compared to control.

To further investigate the relationship between follicular atresia, granulosa cell apoptosis, and ferroptosis, qRT-PCR was used to detect the expression levels of apoptosis marker genes Bcl-2 associated X protein (BAX), B-cell lymphoma-2 (*BCL2*), tumor protein p53 (*p53*), and cysteinyl aspartate specific proteinase 3/9 (*caspase3/9*) in the control group and erastin treatment group, and the results showed that the ratio of *BCL2/BAX* was significantly decreased, while the expression level of *p53* was significantly increased. Ferroptosis is characterized by the accumulation of lipid ROS and MDA and the inhibition of *GPX4* and *SLC7A11*. *GPX4* is an enzyme required to clear lipid ROS and plays a crucial role in the cellular antioxidant system [13]. When *GPX4* is inhibited, lipid peroxides expand and accumulate, inducing ferroptosis [14]. It has been reported that if the function of *SLC7A11* is inhibited, cysteine may break down, resulting in damage to the *GPX4* antioxidant defense axis [15]. Therefore, we detected the expression levels of ferroptosis marker genes in the control group and the treatment group, and the expression levels of *GPX4*, *SLC7A11*, and *FTH* decreased, while the expression levels of *ACSL4* and nuclear receptor coactivator 4 (*NCOA4*) increased (Figure 2A). The induction mechanism of the ferroptosis inducer erastin is related to ROS and iron-dependent signaling. Erastin inhibits voltage-dependent anion channels and accelerates oxidation, leading to the accumulation of endogenous reactive oxygen species. A previous study found that ferroptosis was associated with the formation of atretic follicles, so we treated follicles with erastin and then simulated the atretic follicle environment. To further demonstrate the inhibitory effect of erastin on granulosa cell proliferation, we performed CCK-8 tests on granulosa cell proliferation after treatment with a 10 μM concentration of erastin, and the results showed that significant differences in cell proliferation began to appear at 12 h after treatment. The cell viability was significantly reduced after treatment with erastin (Figure 2B). To further investigate the effect of ferroptosis on the proliferation of ovarian granulosa cells, 5-ethynyl-2′-deoxyuridine (EDU) staining was performed on granulosa cells after treatment with erastin. The results showed that the number of cell proliferation in the control group was significantly higher than that in the treatment group (Figure 2C,D). The Mitotracker Red CMXRos (Mitotracker) staining results of mitochondrial activity showed that the mitochondrial activity of the cells in the erastin treatment group was also inhibited, and the fluorescence intensity decreased compared with the control group (Figure 2E,F). Consistently, ROS staining reflected reactive oxygen species, indicating a significant increase in ROS in treated cells compared to control cells (Figure 2G,H). At the same time, we conducted in vitro experiments on granulosa cells to confirm the previous results; compared with the control group, the expression levels of *FTH*, *FTL*, *GPX4*, and *SLC7A11* in erastin-treated granule cells were significantly reduced, and the expression levels of NADPH oxidase 4 (*NOX4*) were significantly increased (Figure 2I). The Western blot (WB) results showed that the protein contents of *ACSL4* and Transferrin receptor (*TFRC*) were significantly increased in the erastin treatment group, while the protein contents of *GPX4* and *FTH* were significantly decreased (Figure 2J). The above results showed that the erastin treatment of granulosa cells promoted ferroptosis and inhibited granulosa cell proliferation.

### 2.3. Differential Expression of tRFs in Healthy Follicles after Treatment with Ferroptosis Inducer Erastin

**Figure 3:**
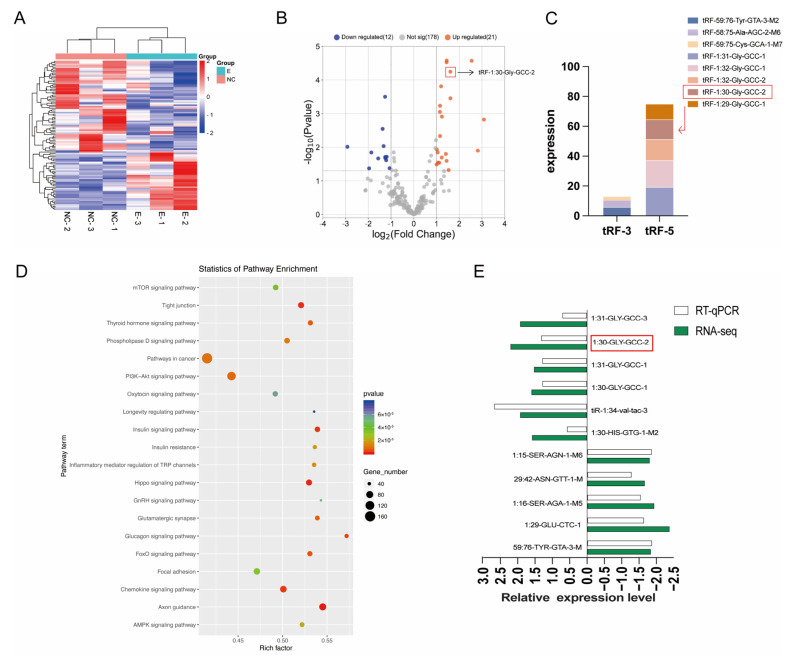
tRFs number statistics and clustering heat maps of differences in sequencing between healthy follicles and inducer-treated follicles. (**A**) tRFs clustering heat map of sequencing data differences. (**B**) Sequencing data variance tRFs volcano map. (**C**) The abundance values of tRF-3 and tRF-5 were analyzed. (**D**) KEGG enrichment analysis. (**E**) qRT-PCR results verified the sequencing data.

We used qRT-PCR to analyze the differential expression of tRFs between the control group (DMSO group) and erastin treatment group after sequencing. DESeq2, as a negative binomial distribution-based method, is suitable for the differential expression analysis of RNA-seq data. Therefore, we used the DEseq2 method to identify the differentially expressed tRFs in the two groups. Hierarchical clustering showed that samples from the erastin treatment group were clustered together (Figure 3A). Using the definition criteria of a fold change ≥ 1.5 and *p*-value < 0.05, 35 differentially expressed tRFs were identified in the treatment group, of which 21 were up-regulated in the treatment group and 12 were down-regulated in the treatment group (Figure 3B). tRF-3 and tRF-5 in the results of sequencing data were classified. We found that the content of tRF-1:30-GLy-GCC-2 was high and its sequence was conserved, so we chose it as the object of study (Figure 3C). We performed a KEGG pathway enrichment analysis for differentially expressed genes. Through the KEGG analysis, enrichment in the Hippo signaling pathway was found (Figure 3D). RT-qPCR was performed on five randomly selected control groups and treatment groups to verify RNA-seq data. The two methods showed similar trends in tRFs expression changes (Figure 3E).

### 2.4. MAPK1 Is a Positional Target of tRF-1:30-Gly-GCC-2

**Figure 4:**
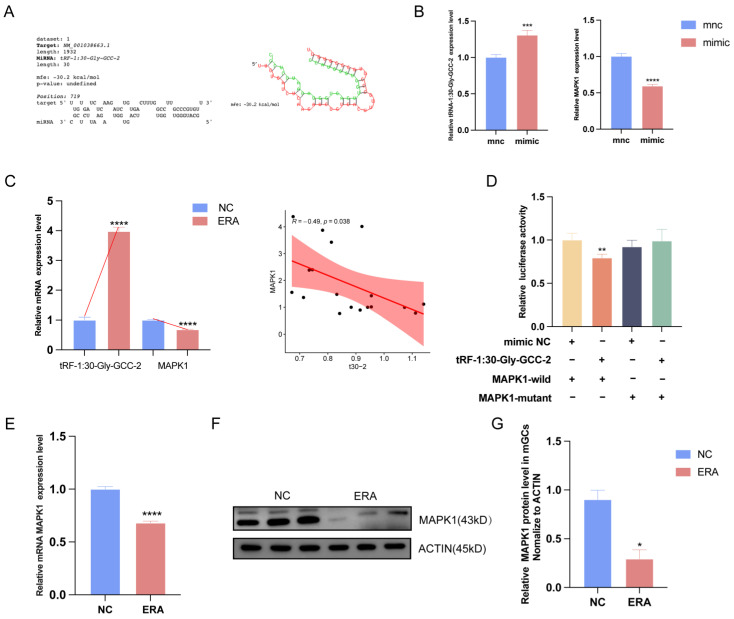
MAPK1 is the target of tRF-1:30-Gly-GCC-2. (**A**) The binding site of tRF-1:30-Gly-GCC-2 to MAPK1 was predicted. (**B**) The expression of *MAPK1* in control group and overexpression group. (**C**) Correlation analysis of tRF and *MAPK1*. (**D**) Dual luciferase reports detected tRF-1:30-Gly-GCC-2 binding to *MAPK1*. (**E**) The expression level of *MAPK1* in the control group and erastin group was detected by qRT-PCR, and the results of WB (**F**,**G**) were consistent with them. Data represented the mean ± SEM of at least three biological replicates. * *p* < 0.05, ** *p* < 0.01, *** *p* < 0.001 and **** *p* < 0.0001, compared to control.

After analyzing the data, we found that the expression level of tRF-1:30-Gly-GCC-2 was high and the trend was consistent in follicles and cells. So, we used RNAhybrid to predict the target of tRF-1:30-Gly-GCC-2 (Figure 4A) and found that the expression of *MAPK1* decreased after the overexpression of tRF-1:30-Gly-GCC-2 (Figure 4B). In conclusion, the expression level of tRF-1:30-Gly-GCC-2 was negatively correlated with *MAPK1* (Figure 4C). As the integration point of various biochemical signals, *MAPK1* is involved in various cellular processes such as proliferation, differentiation, transcriptional regulation, and development [16]. Our results suggest that tRF-1:30-Gly-GCC-2 may target the *MAPK1* gene in granular cells. Therefore, we chose *MAPK1* for further study. To further confirm whether tRF-1:30-Gly-GCC-2 targets *MAPK1*, we performed a dual luciferase reporter gene assay. Compared with the mnc (mimic control group) in granular cells, luciferase activity levels were significantly reduced when *MAPK1-WT* (*MAPK1* wild type) responded to the tRF-1:30-Gly-GCC-2 mimic. However, the tRF-1:30-Gly-GCC-2 mimic did not alter luciferase activity in the *MAPK1-MUT* (*MAPK1* mutant) (Figure 4D). MAPK pathway research is mainly focused on cancer and immune and inflammatory diseases, regulating cell apoptosis and other biological processes [17]. In order to explore the role of *MAPK1* in the process of ferroptosis in granular cells, this study first detected the expression level of *MAPK1* in granular cells treated with erastin. The RT-qPCR results showed that the expression level of *MAPK1* was significantly decreased in the treated cells, and the WB results were consistent with the quantitative results (Figure 4E–G). The results showed that tRF-1:30-Gly-GCC-2 inhibited the relative luciferase activity of the reporter gene by binding the *MAPK1* 3′ untranslated region sequence. These results indicated that *MAPK1* was a direct target gene for tRF-1:30-Gly-GCC-2 aggregation in granular cells.

### 2.5. Overexpression of tRF-1:30-Gly-GCC-2 or MAPK1 Knockdown Inhibited Granulosa Cell Proliferation

**Figure 5:**
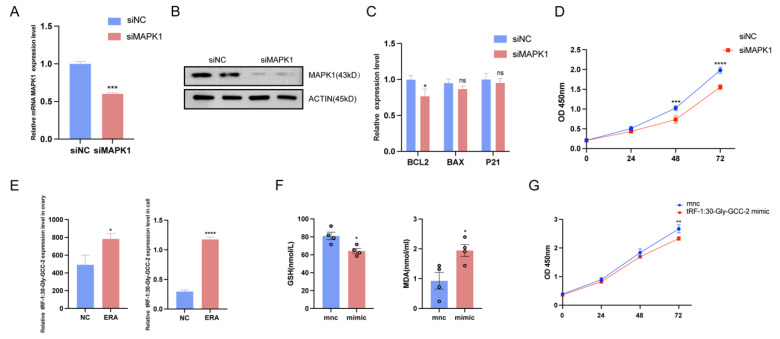
Overexpression of tRF-1:30-Gly-GCC-2 or *MAPK1* knockdown inhibited granulosa cell proliferation. (**A**) The expression of *MAPK1* in siNC group and siMAPK1 group. The results of WB (**B**) are consistent with those of PCR. The expression of tRF-1:30-Gly-GCC-2 was observed in follicular and granulosa cells and erastin group. (**C**) Apoptosis marker genes in siMAPK1 group in granulosa cells were detected by qRT-PCR. (**D**) CCK-8 detected the viability of cell knockdown *MAPK1*. (**E**) Expression of tRF-1:30-Gly-GCC-2 in follicle and granulosa cells treated with erastin. (**F**) Contents of GSH and MDA in mnc group and tRF-1:30-Gly-GCC-2mimic group. (**G**) CCK-8 detected the viability of cells overexpressing tRF-1:30-Gly-GCC-2. Data represented the mean ± SEM of at least three biological replicates. ns > 0.05, * *p* < 0.05, ** *p* < 0.01, *** *p* < 0.001 and **** *p* < 0.0001, compared to control.

To further verify whether tRF-1:30-Gly-GCC-2 regulates ferroptosis through *MAPK1*, we knocked down *MAPK1*; the expression of *MAPK1* was significantly decreased (Figure 5A), and at the same time, we also detected the protein expression of *MAPK1* after knockdown, and the results were consistent with the quantitative results (Figure 5B). In addition, we found that the apoptosis marker gene *BCL2* decreased significantly, and the expression levels of *BAX* and tumor protein p21 (*p21*) showed no significant difference compared with the control group (Figure 5C). We then examined cell viability in the control group and after *MAPK1* was knocked down. The CCK-8 results showed that after *MAPK1* was knocked down, cell proliferation began to be inhibited at 48 h, and cell viability was significantly reduced (Figure 5D). Our results showed that the expression of tRF-1:30-Gly-GCC-2 was elevated in healthy follicular and ovarian granulosa cells treated with erastin (Figure 5E). At the same time, we detected the contents of intracellular GSH and MDA indexes after the overexpression of tRF-1:30-Gly-GCC-2 and found that the contents of GSH decreased while the contents of MDA increased (Figure 5F). This was consistent with the results after treatment with erastin. In order to explore whether tRF-1:30-Gly-GCC-2 in the sequencing results plays a regulatory role in ovarian granulosa cell death, the overexpression of tRF-1:30-Gly-GCC-2 in granulosa cells was conducted to explore whether tRF-1:30-GLy-GCC-2 played an important role. The results showed that the overexpression of tRF-1:30-Gly-GCC-2 significantly inhibited granulosa cell proliferation for 72 h (Figure 5G). These results indirectly demonstrated that tRF-1:30-Gly-GCC-2 could inhibit cell proliferation, and the results were consistent with the down-regulation of *MAPK1*.

### 2.6. tRF-1:30-Gly-GCC-2 Inhibits Granular Cell Proliferation and Promotes Ferroptosis by Targeting MAPK1

**Figure 6:**
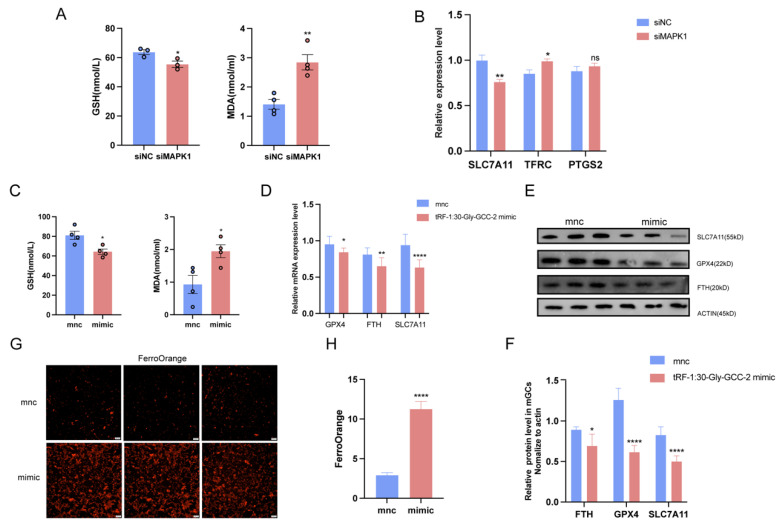
tRF-1:30-Gly-GCC-2 promotes ferroptosis in granular cells through *MAPK1*. (**A**) The contents of GSH and MDA in cells after transfection with siMAPK1. (**B**) The expression of iron sag marker genes *SLC7A11*, *TFRC*, and *PTGS2* after MAPK1 knockout was detected by qRT-PCR. (**C**) Contents of GSH and MDA in mnc group and tRF-1:30-Gly-GCC-2mimic group. (**D**) The expression of ferroptosis marker gene *GPX4*, *FTH*, and *SLC7A11* after overexpression of tRF-1:30-Gly-GCC-2 was detected by qRT-PCR. The results of WB (**E**,**F**) were consistent with them. (**G**,**H**) FerroOrange (200 μm) content of iron ion probe in control group and overexpression group. Data represented the mean ± SEM of at least three biological replicates. ns > 0.05, * *p* < 0.05, ** *p* < 0.01 and **** *p* < 0.0001, compared to control.

These results indicate that *MAPK1* plays an important role in the pathogenesis of ferroptosis in granular cells. The levels of GSH and MDA in granulosa cells were detected after *MAPK1* was knocked out. The results showed that the intracellular GSH content decreased, and the MDA content increased after *MAPK1* knockdown (Figure 6A). Further, the expression of ferroptosis marker *SLC7A11* was significantly down-regulated, and the expression of *TFRC* was significantly up-regulated (Figure 6B). At the same time, the contents of intracellular GSH and MDA indexes after the overexpression of tRF-1:30-Gly-GCC-2 were analyzed, and it was found that the contents of GSH decreased while the contents of MDA increased (Figure 6C). We detected the expression levels of ferroptosis marker genes. Compared with the control group, the expression levels of *GPX4*, *SLC7A11*, and *FTH1* were significantly decreased after the overexpression of tRF-1:30-Gly-GCC-2 (Figure 6D). At the same time, the WB method was used to detect the protein expression levels in the two groups, and the protein contents of *GPX4*, *SLC7A11*, and *FTH1* were significantly decreased after the overexpression of tRF-1:30-Gly-GCC-2 (Figure 6E,F). In addition, FerroOrange is a fluorescent probe for the specific detection of unstable Fe^2+^. Once it reacts with Fe^2+^, a red fluorescent substance is formed. Fe^3+^ or divalent metal ions other than iron do not enhance its fluorescence. Therefore, we detected the fluorescence intensity of FerroOrange in the control group and in granulocyte cells after the overexpression of tRF-1:30-Gly-GCC-2, and it was found that the FerroOrange content in the cells overexpressing tRF-1:30-Gly-GCC-2 was higher than that in the control group (Figure 6G,H). It was shown that tRF-1:30-Gly-GCC-2 inhibits granular cell proliferation and promotes ferroptosis by targeting *MAPK1*.

## 3. Discussion

The occurrence of atretic follicles may lead to irregular menstruation, ovulation disorders, and the formation of cysts, which is an important cause of female infertility [18]. Although the molecular mechanism of ovarian dysfunction and atresia remains to be elucidated, it has been reported that granulosa cell apoptosis is closely related to atresia.

The proliferation, development, and apoptosis of GCs play an important role in the regulation of ovarian function and the occurrence of follicular atresia. Apoptosis and ferroptosis are two different forms of cell death [19]. Apoptosis is an unprogrammed form of death and one of the most studied methods of cell death [20]. It is triggered by two different pathways; one is the exogenous death receptor pathway and the other is cell death induced by Bcl family proteins [21]. These pathways lead to a reduction in cell size, an increase in cytoplasmic density, and then a series of changes that eventually divide the apoptotic cell remains into several apoptotic bodies [22]. In contrast to apoptosis, ferroptosis is an iron-dependent injury involving membrane lipids, and many metabolic pathways involving iron, lipids, and amino acids control the sensitivity of cells to ferroptosis [23]. The concept of ferroptosis was first proposed by Dr. Brent R. Stckwell of Columbia University in 2012 [24]. It is a new type of iron-dependent programmed cell death, which is different from apoptosis, cell necrosis, and autophagy [25,26]. It was reported in 2012–2014 that the pathway of inducing cell ferroptosis is the excessive accumulation of lipid peroxides up to well-known levels, and finally inducing cell ferroptosis [27]. Therefore, a large number of studies initially focused on the decline of *GPX4* activity leading to glutathione depletion, the inability of lipid peroxides to metabolize through GPX-catalyzed glutathione reductase reactions, and ultimately resulting in the oxidation of diferric ions and the production of reactive oxygen species that ultimately promote the occurrence of cell ferroptosis [28].

GSH is an important antioxidant in the cell, which can maintain the REDOX balance in the cell, clear the free radicals in the cell, reduce the oxidative stress of the cell, and may participate in the regulation of the concentration of iron ion in the cell, and its content is negatively correlated with the occurrence degree of ferroptosis [29]. Xia et al. found that erastin induced cell death and reduced levels of the intracellular antioxidant GSH [30]. MDA is the final product of ferroptosis in cells. The excessive accumulation of MDA will lead to severe membrane peroxidation, and its content is positively correlated with the degree of ferroptosis [31]. Ovarian granulosa cells treated with follicular fluid in mice with endometriosis showed abnormal signaling pathways of cell senescence and ferroptosis [32]. Ferrous ions, ROS, and lipid peroxide (LPO) were detected. Increased levels of LPO and MDA, and shorter and more reconstituted mitochondria were observed in KGN and granulosa cells after follicular fluid treatment in endometriosis [33]. Liu et al. found that MDA content was significantly reduced after the addition of Ferrostatin-1 (Fer-1), a ferroptosis inhibitor, in lipopolysaccharide-induced acute lung injury [34]. In addition, as one of the key markers of ferroptosis in cells, the increase in ferrous ions can increase the level of oxidative stress in the intracellular environment [35]. In this study, we found that the amount of GSH was significantly reduced in the atretic follicles. The content of MDA in the follicular fluid of atretic follicles was significantly higher than that of healthy follicles, and the content of ferrous ions was significantly increased.

In addition, we successfully constructed an artificial model of atretic follicles in vitro, adding a 10 μM concentration of erastin to 4 mm healthy follicles, and found that the treated follicle phenotype was consistent with that of atretic follicles. Moreover, when the same concentration of erastin was added to the granule cells, it was found that the cell vitality and cell morphological changes were significantly decreased after treatment, which was consistent with the occurrence of ferroptosis. Our results are identical to those of Wei et al. [36].

tRNA is a key adaptation molecule for deciphering the genetic code in mRNA translation during protein synthesis [37]. tRFs are an emerging class of small non-coding RNAs produced by the cleavage of mature tRNAs or tRNA precursors. tRF regulates cell viability, differentiation, and homeostasis through a variety of mechanisms [38]. It is now increasingly recognized that trnas display tissue-specific and cell-type-specific expression patterns. Our study aimed to discuss the potential function and mechanism of tRF-1:30-Gly-GCC-2 in the occurrence of ferroptosis in granular cells.

We sequenced the successfully constructed atretic follicle model after erastin treatment and performed KEGG functional enrichment analysis. The Hippo-YAP/TAZ signaling pathway is a novel regulatory mechanism for ferroptosis. Recent studies have found that the Hippo-YAP/TAZ signaling pathway affects the sensitivity of tumor cells to ferroptosis through a variety of extracellular pathways such as cell density, cell contact, cell metabolism, and mechanical signals [39]. In different types of tumor tissues, the Hippo-YAP/TAZ signaling pathway affects the sensitivity of tumor cells to ferroptosis through specific stimulation conditions, ferroptosis targeting proteins, and their molecular mechanisms [40]. It affects the occurrence and development of tumors in urinary, reproductive, digestive, respiratory, and endocrine systems. Previous results showed that ferroptosis was associated with the development of atretic follicles, so we examined apoptosis and the expression of ferroptosis-related marker genes. It was found that the content of tRF-1:30-Gly-GCC-2 was significantly increased in the atretic follicle model. The subsequent overexpression of tRF-1:30-Gly-GCC-2 promoted granulosa cell apoptosis by decreasing the ratio of *Bcl2* to *BAX*, and increased ferroptosis by down-regulating *GPX4* and *SLC7A11*, and up-regulating the expression of *TFRC*, *NOX4*, and *ACSL4* in mGCs cell lines. Indirectly, tRF-1:30-Gly-GCC-2 can promote ferroptosis in granulosa cells.

Regarding the possible mechanism of tRF-1:30-Gly-GCC-2 action, we screened the potential target *MAPK1* of tRF-1:30-Gly-GCC-2. MAPK is an important transmitter of signals from the cell surface to the interior of the nucleus. Liu et al. obtained ferroptosis Differential Expressed Genes (DEGSs) by comparing Pondus Hydrogenii (PH) with the corresponding gray matter and contralateral white matter [41]. Through the FerrDb online tool, the DEGS is further divided into ferroptosis drivers, ferroptosis inhibitors, and ferroptosis markers [41]. Among them, *MAPK1* appears in the database as the driving factor of ferroptosis. Subsequently, by detecting the changes of granular cells in oe-tRF-1:30-Gly-GCC-2 and siMAPK1 groups, the results showed that the probability of ferroptosis in granular cells in siMAPK1 group was increased.

## 4. Materials and Methods

### 4.1. Dissection, Culture, and Treatment of Porcine Follicles

#### 4.1.1. Preparation of Follicle Samples

The porcine follicles in this experiment were collected from a slaughterhouse in Chengdu, Sichuan Province, and brought back to the laboratory within 1 h after slaughter for stripping treatment and subsequent preparation of RNA-seq samples.

After the blood stains were cleaned with 38 ± 1 °C normal saline, the sow ovaries were placed in 1×PBS containing 1% streptomycin mixture and brought back to the laboratory at 38.6 °C. After taking the ovaries back to the laboratory, they were washed once with 75% ethanol (AR grade) and then twice with a saline solution. After washing, the ovaries in the bottle were picked up with long forceps and placed in a petri dish with PBS solution for stripping treatment.

A scalpel was used to cut the cortical part of the surface of the ovary, followed by the use of sharp forceps to tear the cortical portion of the surface of the follicle and remove the connective tissue from the root, thus obtaining a complete follicle. The follicles were placed in a culture dish and then taken to a super-clean table and washed twice with DMEM/F12 medium containing 1% streptomycin mixture solution, then placed in a high-temperature disinfected culture dish and cultured with the same medium in a cell incubator at 37 °C and 5% CO_2_.

#### 4.1.2. Handling of Follicle Samples

The follicles were gently added to the cell culture dish with short tweezers. A total of 1 µL of DMSO per mL of medium was added to the control group, and 1 µL of erastin (diluted with DMSO according to the instructions) with a concentration of 10 μM per mL of medium was added to the experimental group, and the samples were collected after 24 h of treatment.

The treated follicle samples were drained of medium on the surface using filter paper, and the follicles were frozen in liquid nitrogen and placed in a pre-cooled cryostorage tube. After collecting the samples, the cryostorage tube was stored in an ultra-low temperature refrigerator at −80 °C.

### 4.2. Cell Culture

The granule cell line was purchased from Pricells. The granular cells were cultured with DMEM/F12 medium containing 10% FBS of 1% triple antibody, and transferred to the incubator, and the culture medium was changed after the cell adhesion was observed the next day. All cells were cultured at 37 °C in a 5% CO_2_ cell culture chamber. Cells were digested with trypsin and spread in cell culture plates at a density of 100,000 cells per mL, and the experimental treatment was started when the cell density grew to 80%. An erastin concentration of 10 μM was injected into mouse granular cell lines for 24 h.

### 4.3. RNA Extraction and Reverse Transcription

RNA was extracted according to RNAiso (Takara) standard, and cDNA was prepared using PrimeScript TM RT Reagent Kit with gDNA Eraser (Takara, Dalian, China).

### 4.4. Real-Time Fluorescent Quantitative PCR

The fluorescence quantitative upper plate was placed in the internal groove of the machine, the machine lid was closed, and the amplification program was set as follows: ① for 94 °C for 120 s pre-denaturation, ② for 94 °C for 30 s denaturation, ③ for 60 °C for 30 s annealing, and ④ for 72 °C for 30 s extension, in which ②–④ cycles were performed 40 times, and then the temperature was lowered to 4 °C to end the amplification detection.

### 4.5. Assessment of Reduced Glutathione (GSH), Malondialdehyde (MDA), and Iron Levels

In our study, to assess ferroptosis levels in different groups, GSH, MDA, and Fe^2+^ levels were measured in each group. The reduced GSH test kit (Elabscienc, Houston, TX, USA, article number: E-BC-K030-M), MDA test kit (Elabscienc, article number: E-BC-K028-M), and Fe^2+^ test kit (Elabscienc, article number: E-BC-K773-M) evaluates GSH concentration, MDA concentration, and Fe^2+^ concentration according to manufacturer’s instructions.

### 4.6. Immunofluorescence

#### 4.6.1. EDU

In this experiment, 96-well plates were used to culture ovarian granule cells. The EdU solution (reagent A) was diluted with cell complete culture medium at a 1000:1 ratio to prepare an appropriate EdU medium of 50 μM. A total of 100 μL 50 μM EdU medium was added to each well, incubated for 2 h, and then the medium was discarded. A total of 1×PBS was added, the dye was washed 1–2 times, 50 μL cell fixative containing 4% paraformaldehyde was added into each well, the cell plate was placed in a flat refrigerator at 4 °C overnight to fix the cells, and then the fixative was thrown away. A total of 50 μL 2 mg/mL glycine per well was added and incubated at room temperature for 5 min; the supernatant was discarded, then 1×PBS was added and washed 1–2 times. A total of 150 μL puncture solution (0.5% TritonX-100 PBS) was added to each well, incubated for 10 min, and cleaned once with PBS. A total of 100 μL of 1× APOLo@ dyeing reaction solution was added to each well (according to the kit instructions, use on the spot), incubated at room temperature, and placed on the decolorizing table for 60 min; then, the dyeing reaction solution was discarded. Reagent F was diluted in a ratio of 100:1 in deionized water, and an appropriate amount of Hoechst33342 working liquid was prepared and stored away from light. A total of 100 μL Hoechst33342 working liquid was added to each well and incubated in a decolor shaker at dark room temperature for 30 min. The staining reaction liquid was discarded, 1×PBS was added, washed twice, and observed under a microscope; then, photos were taken.

#### 4.6.2. Mitotracker

A tube of 50 μg MitoTracker powder was added with 94.06 microliters of DMSO to form a 1 mM storage solution for use. The storage solution was diluted to 500 nM with serum-free medium and then added to the cell culture plate. After incubation for 30 min, the cells were cleaned twice with preheated 1×PBS. They were observed under a fluorescence microscope.

#### 4.6.3. ROS

The probe was diluted with 1:1000 serum-free medium and added to the cell culture plate. The cell plate was incubated in the incubator for 30 min, and then the medium was replaced with fresh medium. They were observed under a fluorescence microscope.

### 4.7. Cell Viability Assay

CCK-8 reagent was added to a 96-well cell culture plate, 10 μL CCK-8 reagent was added to the 100 μL medium, and the cell culture plate was incubated in the incubator for 2 h, and the absorbance of 450 nm was determined by enzyme labeling.

### 4.8. Western Blot

We added 100 μL RIPA lysate (containing PMSF and phosphatase inhibitors) to 106 cells, detected the protein concentration with a BCA kit (Beyond Biotech Co., Ltd., Xinchang, China), and deplasticized the protein with 5× SDS Buffer. The protein was denatured at 95 °C for 10 min. After treatment, the protein was subjected to SDS PAGE electrophoresis until the blue band of bromophenol extended to the bottom of the gel. The methanol-activated PVDF membrane was covered on the gel for the transmembrane experiment, incubated at 200 mA for 2 h, and then incubated with primary and secondary antibodies. Finally, the ECL color developer was used to expose the strip, and ImageJ 2.3.0/1.53q was used to calculate the gray level of the strip.

### 4.9. Criteria for Determining the Degree of Follicular Atresia

The peeled fresh follicles were absorbed with filter paper to remove the residual PBS containing the 1% green streptomycin mixed solution from the outside of the follicles, and the follicles of 4–5 mm were selected for subsequent experimental treatment by measuring the diameter of the follicles with vernier calipers. The blood vessels on the surface of the healthy follicles were abundant, transparent, and pink in color. The primary atretic follicle showed a small number of blood vessels, which were pale pink in color and poor in transparency. There was no blood vessel distribution on the surface of the atretic follicle, and the internal follicle was cloudy.

### 4.10. Statistical Analysis

All data were analyzed using the GraphPad Prism 9.0 software and expressed as means ± standard deviation (SD). A one-way analysis of variance (ANOVA) was then used to determine whether there were any significant differences between the means of two groups, with the significance threshold set at *p* ≤ 0.05.

## Data Availability

The datasets used or analyzed during the current study are available from the corresponding author upon reasonable request.
